# Epigenetics and Metabolism Reprogramming Interplay into Glioblastoma: Novel Insights on Immunosuppressive Mechanisms

**DOI:** 10.3390/antiox12020220

**Published:** 2023-01-18

**Authors:** Filippo Torrisi, Simona D’Aprile, Simona Denaro, Anna Maria Pavone, Cristiana Alberghina, Agata Zappalà, Rosario Giuffrida, Lucia Salvatorelli, Giuseppe Broggi, Gaetano Giuseppe Magro, Vittorio Calabrese, Nunzio Vicario, Rosalba Parenti

**Affiliations:** 1 Department of Biomedical and Biotechnological Sciences, Section of Physiology, University of Catania, 95123 Catania, Italy; 2 Department of Medical, Surgical Sciences and Advanced Technologies “G.F. Ingrassia”, Anatomic Pathology, University of Catania, 95123 Catania, Italy; 3 Department of Biomedical and Biotechnological Sciences, Section of Biochemistry, University of Catania, 95123 Catania, Italy

**Keywords:** glioblastoma, epigenetics, immunometabolism, tumor microenvironment, immunosuppression

## Abstract

The central nervous system represents a complex environment in which glioblastoma adapts skillfully, unleashing a series of mechanisms suitable for its efficient development and diffusion. In particular, changes in gene expression and mutational events that fall within the domain of epigenetics interact complexly with metabolic reprogramming and stress responses enacted in the tumor microenvironment, which in turn fuel genomic instability by providing substrates for DNA modifications. The aim of this review is to analyze this complex interaction that consolidates several conditions that confer a state of immunosuppression and immunoevasion, making glioblastoma capable of escaping attack and elimination by immune cells and therefore invincible against current therapies. The progressive knowledge of the cellular mechanisms that underlie the resistance of the glioblastoma represents, in fact, the only weapon to unmask its weak points to be exploited to plan successful therapeutic strategies.

## 1. Introduction

Glioblastoma (GBM) is one of the main solid tumors that brings together the main hallmarks of cancer, conferring it a complexity and heterogeneity that make difficult the upmost therapeutical approaches [1]. The abnormal and dysregulated neovascularization associated with its typical pattern, consisting of necrotic foci with surrounding cellular pseudopalisades and microvascular hyperplasia, distinguishes GBM as a neoplasm with the lowest oxygenation levels. Such a characteristic makes GBM resistant to the current treatment protocol that includes surgical resection, radiotherapy and chemotherapy [2,3,4,5].

Metabolic rewiring and immune suppression in GBM are two closely related features, supporting pathogenesis and an aggressive pattern, limiting the efficacy of standard therapies and novel clinical approaches such as immunotherapy [6]. Indeed, the dynamic metabolic condition characterizing GBM profoundly reshapes the features of the tumor microenvironment (TME), thus creating hostile conditions for T cell proliferation and survival, negatively affecting the host immune response [6]. Several lines of evidence show that GBM progression is closely related to the interaction that tumor cells establish with other cell populations in the TME, which adopt mechanisms to avoid being detected and killed by immune cells [7]. In the same way that under physiological conditions, glial cells can influence stromal cells’ behavior, GBM cells can similarly mediate the homing, the recruitment and the differentiation of infiltrating cells in paracrine fashion or via a direct cell–cell contact [8,9,10,11,12].

In addition to glucose consumption and significant impairment in cytolytic activity and cytokine secretion due to TME acidification, several additional pathways in GBM drive immunosuppressive mechanisms [1]. In particular, aberrant lipid metabolism and specifically high free fatty acid levels closely modulate the immune response in the TME [13,14,15]. Moreover, like many other cancers, GBM also exhibits “glutamine addiction”. Glutamine serves as the major contributor to cell growth and energy production after it is converted into glutamate via glutaminase, and then into α-ketoglutarate (α-KG) via glutamate dehydrogenase or several aminotransferases [16]. Mesenchymal GBM, which is the most aggressive molecular subtype, reported a significantly higher uptake of glutamine to generate glutamate-derived metabolites such as α-KG to produce ATP [17,18]. Glutamine deprivation in the TME determines the ideal conditions for immunosuppression due to the production of interleukin-23 (IL-23), interleukin-10 (IL-10) and transforming growth factor-beta (TGF-β), stimulating immune-suppressive regulatory T cells and suppressing cytotoxic cells [19]. An additional detrimental factor to this dynamic metabolic condition is the onset of stress responses, such as hypoxia or anoxia, caused by the lack of an appropriate vascular system in the tumor core. A hypoxic TME dramatically reshapes the transcriptional landscape, which profoundly impacts metabolic networks [20,21]. Indeed, in hypoxic conditions, the dimerization of hypoxia inducible factors (HIFs) determines the transcriptional activation of several genes involved in metabolic reprogramming and immunosuppression [22,23]. Hence, immunometabolism can be considered an integrated hallmark of cancer cells that ensures their ability to escape immune system surveillance by masking DNA damage signaling factors for cellular defense activation or by releasing factors that prevent immune system aggression. Therefore, this condition fosters the path of neoplastic transformation [24,25].

Despite metabolic reprogramming being largely depicted as a prominent factor in restructuring the TME immune system of GBM, uncovered mechanisms between metabolism and immune evasion should be explored further, offering new research perspectives. In particular, the high heterogeneity and complexity of GBM suggest a critical influence of epigenetic factors including DNA methylation, histone modifications and microRNA (miRNA) post-transcriptional gene regulation, which remodel the genetic composition of the tumor and the interaction between immune cells and TME metabolic niches, thus favoring the escape from immune destruction [26,27,28,29,30,31].

**Figure 1:**
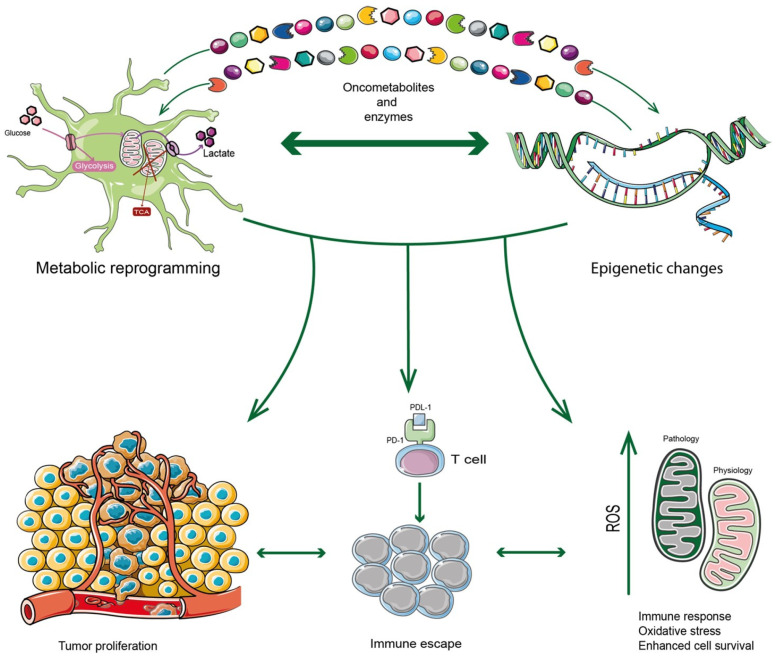
GBM tumor cells are highly inclined to find the metabolic adaptation to survive in hostile conditions including attacks by the immune system. In this regard, their metabolic reprogramming is induced by epigenetic changes that determine the transcription of enzymes or factors belonging to several metabolic pathways. At the same time, enzymes or metabolites can also trigger epigenetic changes. The crosstalk between metabolism and epigenetics can be considered a bridge that leads to immunosuppression mechanisms, generating GBM tumor progression in a complex TME characterized by oxidative stress as well.

In recent years, the epigenetic mechanisms influencing the TME in different cancers, including GBM, have acquired an expanding field of interest. Huo et al. examined glycolysis and gluconeogenesis enzymes by epigenetic modifications including miRNA, circular RNA and long non-coding RNA in HIFs regulation. They also reported how glucose metabolites, tricarboxylic acid (TCA) cycle, lipids and amino acids, as well as metabolites produced by gut microbiota, provide substrates for epigenetic modifications [32]. Wu et al. reported the impact of epigenetic modifications on the evolution of adaptive resistance to therapy in GBM [33]. The role of chromatin and epigenetic dysregulation have also been reported to promote glioblastoma stem cells (GSCs), which represent therapy-resistant reservoirs in GBM [26]. Epigenetic regulators in response to treatments were evaluated in shaping the phenotypic heterogeneity of GBM, reporting a variable balance between pre-existing and adaptive resistant cells, trying to clarify mutable adaptation to treatment [34]. Markouli et al. summarized main drugs targeting the epigenetic and metabolic interplay in gliomas from preclinical and clinical studies [35]. The crosstalk between epigenetics and metabolism was recently reviewed in GBM, showing its dual role in promoting and inhibiting the activity of metabolic pathways [35]. However, despite these advanced investigations, the underlying epigenetic mechanisms have not yet been fully elucidated in driving the immunosuppressive state of GBM. In the intricate TME of GBM, a bidirectional linkage of metabolism and epigenetic modification is responsible for metabolic reshaping and histones/DNA modifications. Epigenetic regulation modulates the expression of metabolic enzymes, which ultimately affect overall tumor metabolism (Figure 1).

This review aims to provide an insight into the epigenetic regulation and metabolism interaction in establishing an immune suppressive TME towards GBM aggressiveness, highlighting potential therapeutic targets.

## 2. Epigenetics and Metabolism Interplay

The epigenetic theories were firstly introduced in 1942 by C. Waddington, who coined the term and defined it as a “branch of biology which studies the causal interactions between genes and their products which bring the phenotype into being” [36]. Over time, evidence has increasingly converged to define epigenetics as the modifications of gene expressions mitotically and meiotically hereditable without involving modifications of gene sequences [37]. Inside the complex world of epigenetic regulations, the pre-eminent portion is assumed by the chromatin remodeling processes that modify the accessibility to the regions of the genome, also including the non-coding sequences [38]. Most of the DNA in eukaryotic cells is packed into nucleosomes, where it is wrapped around a core of eight histone proteins, which are the most abundant proteins associated with genomes. They consist of two dimers of H2A and H2B associated with a tetramer of H3 and H4, while H1 joins adjacent nucleosomes working as histone linker [39]. Histones are mostly composed of positively charged amino acids including up to 20% lysine, arginine and in minor quantities serin, which generate hydrogen bonds with the oxygen atoms of the phosphodiester of DNA. Nucleosome also contains amino-terminal tails which are not necessary for DNA winding, but represent the fulcrum of substantial modifications capable of changing the function of the nucleosome thanks to phosphorylation, acetylation and methylation of amino acid residues. Modifications of the histone tails can form a histone code that can be encoded by proteins involved in gene expression, repriming or activating specific genes [40].

Epigenetic regulation at the DNA level depends on dynamic interaction in nucleosomes through ATP-dependent remodeling of the histone octamer. Furthermore, the dynamism of histone modifications depends on the action of specific enzymes, including acetyltransferases and deacetylases, which catalyze the addition or removal of acetyl groups on histones, respectively, and methyltransferases and demethylases, which add or remove methyl groups. DNA methylation refers to the covalent bond of a methyl group supplied by S-adenosyl methionine (SAM) to the fifth position of the cytosine ring (5-methylcytosine) or the sixth position of adenine (6-methyladenine), catalyzed by the enzyme DNA methyltransferase (DNMT) [41]. 5-methylcytosine is the most prevalent form of DNA methylation in eukaryotes and occurs predominantly on the cytosines preceding the guanines, the so-called CpG sites or islands. Furthermore, methylated cytosines can be oxidized to 5-hydroxymethylcytosine by ten–eleven translocation enzymes (TET), even if these are less than 5-methylcytosine. However, CpG sites are not always associated with methylation site, as many CpG islands remain constitutively protected from DNA methylation [42]. Other complex mechanisms of epigenetic regulation add to the structural modifications of the DNA–nucleosome interaction. For instance, the addition of acetyl groups changes the conformation of the DNA, making the binding of specific transcriptional complexes more effective, as in the case of bromodomain. Likewise, methylated sites can also be more easily recognized by proteins that act as repressors [43].

The accumulation of genomic instability and copy number alterations lead to genetic and epigenetic changes and transcription disorders, exerting a key role in the development of several tumors, including GBM [44,45]. A critical role in this process is represented by genomic changes induced by copy number variation and single nucleotide mutations and the following transcriptional dysregulation [45]. In this context, the methylation of O6-methylguanine DNA methyltransferase and the following repression of gene expression induce a greater chemoresistant ability to temozolomide, the elected therapy for GBM [46]. Moreover, ubiquitination of histone H2A, which interacts with epidermal growth factor receptor (EGFR), has also been described to control GBM resistance to senescence [47].

Within the complex TME of GBM, chromatin modifications can directly or indirectly be influenced by the activity of metabolic enzymes, metabolites and cofactors. Metabolites such as acetyl-CoA, ATP and SAM are precursors of acetylated, phosphorylated and methylated histones, respectively, while NAD^+^ acts as a cofactor for sirtuin (SIRT1) deacetylase [48]. In GBM, the activity of isocitrate dehydrogenase (IDH) represents a typical example of the relationship between epigenetics and metabolic reprogramming. Firstly, it is worth noticing that from the new 2021 WHO CNS tumor classification, IDH-mut term has been abolished for GBM; in fact, GBM IV grade was only considered as IDH-wt, whereas IDH-mut was limited to astrocytomas, including low-grade glioma [49,50,51]. However, epigenetic changes may drive the progression of IDH-mut low-grade gliomas to high-grade GBM; thus, the role of IDH-mut subtypes should be addressed in this scenario [52,53]. For this reason, here and after in this review we mention IDH-mut by implying that it refers to low-grade forms of glioma.

When mutated, IDH has been associated with tumorigenesis due to the change in enzymatic function. While IDH-wild type (IDH-wt) converts isocitrate and NADP^+^ to α-KG and NADPH, IDH-mutated (IDH-mut) resides in the catalytic pocket and results in a neo-enzymatic activity: α-KG + NADPH → 2-hydroxyglutarate (2-HG) + NADP [54]. This neo-reaction in IDH-mut cells may also use α-KG derived from glutamine, which is converted to glutamate by glutaminase and further metabolized to α-KG [55]. Innate metabolic dysregulation also occurs including L-2- or D-2-hydroxyglutaric aciduria production due to non-functional enzymes, which metabolizes L- or D-2-HG [56]. IDH represents the major pathway for cellular NADPH generation in most tissues, together with the TCA and pentose phosphate pathway (PPP). NADPH is an essential reducing factor that controls cellular defense mechanisms against oxidative damage through glutathione (GSH) reduction by GSH reductase, a crucial antioxidant that acts as a cofactor to reduce hydrogen peroxide. The classification of GBM tumor subtypes highlighted an interesting aspect related to the IDH mutated state and the methylation profile: the pro-neural subtype and the lower grade forms of astrocytoma share an IDH status with a high methylation profile defined glioma CpG island methylator phenotype [57,58]. The correlation between IDH-mut and methylated phenotype in GBM is a clear example of the mutual dependence between metabolism and epigenetics. In fact, the NADPH-dependent reduction of α-KG to the oncometabolite 2-HG leads to the inhibition of α-KG-dependent dioxygenases due to structural similarities between α-KG and 2-HG. Specifically, TET demethylase activity is inhibited by the presence of 2-HG, blocking the demethylation process. D-2-HG also inhibits histone demethylation by blocking the activity of demethylase such as lysine-specific demethylase (KDM) [54]. It was also reported that IDH-mut induced histone hypermethylation in genomic regions associated with DNA damage response pathways [59]. Likewise, it is possible to consider the other side of epigenetic regulation for IDH-wt, giving that IDH determines a lowering of the methylation profile due to the production of α-KG, which is essential for the demethylation functions of histone demethylase 1 (LSD1) and the lysine-specific JmjC domain containing histone demethylase (JHDM) on histones [60].

In addition to IDH activity, acetylation and succinylation, mediated by Acetyl-CoA and succinil-CoA, respectively, have been described as epigenetic processes driving resistance to targeted therapies [61,62]. Indeed, lysin succinylation leads to changes in many mitochondrial metabolic pathways, playing relevant roles in cell metabolism including glycolysis, fatty acid oxidation, urea cycle and glycolysis [63]. The aberration of these pathways is related to the occurrence of many tumors such as breast cancer, gastric cancer and gliomas. The latter is particularly sensitive to histone H3 succinyltransferase action, which promotes the proliferation and development of this tumor [62]. Moreover, other products of aerobic glycolysis include oncometabolites able to regulate epigenetic processes. Lactate released through monocarboxylate transporters (MCTs) represents an emerging oncometabolite which influences epigenetic mechanisms. Indeed, several lactylation sites on core histones in humans have been identified [64]; radiolabeling carbon atoms, it has been observed that lactate represents a source for generating metabolites of the TCA cycle, converting citrate to acetyl-CoA and increasing histone acetylation [65]. However, lactate also causes an increase in α-KG, triggering the previous epigenetic machinery, illustrating demethylated TET enzyme-dependent processes [66]. Lactate accumulation within cells induces histone lactylation, thus promoting cell proliferation and migration, modulating cell metabolism and promoting tumorigenesis [67,68,69]. In addition, lactic acidosis in the TME promotes a series of mechanisms that alter tumor metabolism and promote oncogenesis [70]. Thus, given the strong crosstalk among metabolism and epigenetic modifications in GBM, it becomes critical to explore the underlying mechanisms of immune escape.

## 3. Epigenetics and Metabolism Reprogramming Interplay in Promoting Immunosuppression

Environmental and internal stressful stimuli, such as bacteria, virus, accumulation of metabolites, dead cells and uncontrolled proliferating cells could disrupt the constancy of steady states in an organism [71]. The innate and adaptive response of the immune system has a key role in maintaining tissue homeostasis, encompassing a plethora of effects beyond the gamut of “self” versus “non-self” interactions [72]. Cancer immunosurveillance, especially for extremely aggressive tumors such as GBM, is even more intricate given the complex network of interactions established between stromal, parenchymal and TME sites [7]. This blends the clear-cut boundary between innate and adaptive response, since physiologically the characters of the two responses closely cooperate in the various defense pathways. However, a distinctive feature of the immune system physiology of the CNS is represented by the tissue-resident microglia cells, that in GBM are known by the names tumor or glioma, associated microglia/macrophages (TAMs) and dendritic cells, which inhabit a major region of the brain parenchyma [73]. Cytokines, chemokines and nitric oxide (NO) are produced by microglia to initiate innate responses with phagocytic and cytotoxic functions, which can trigger additional responses due to the recruiting of soluble factors and peripheral immune cells, including natural killer cells, lymphocytes and macrophages. Moreover, CD4- and CD8-specific T cells for adaptive immune response are also mediated by the activation of microglia, which act as antigen presenting cells (APCs), upregulating MHC and co-stimulatory molecules [74]. A significant influence on the adaptive immune response comes from the meningeal spaces where the immune repertoire is mainly composed of cytokine-secreting CD4 T cells including IL-4, IFN-γ and IL-17 [75]. This evidence supports the change in the view of the CNS as an immune-privileged site; rather, the existence of a TME populated by immune cells dominates the processes of immunosuppression. Indeed, GBM is able to bypass the immunological response of several strategies which do not depend only on the physiology and anatomical aspects of the CNS related to the presence of the protection offered by the BBB, limiting T cell trafficking and infiltration [7]. Certainly, as for most tumors, a reduced immune response is ascribable to the aggressive pharmacological and radiotherapy treatments which cause a depletion of the myeloid components [76]. T cell deficiencies can also result from a senescence process triggered by premature thymic involution in GBM patients [77]. In addition to quantitative defections, T cells have qualitative limitations determined by two interconnected processes, named anergy and exhaustion: the chronic exposure of T cells to antigens and inflammatory signals convoy them in a hyporesponsive or anergic state leading to exhaustion, which impairs T cell activation and downregulates cytokines [78]. T cell exhaustion is also supported by the immunosuppressive phenotype of APCs due to the expression of inhibitor-immune checkpoints, such as PD-1 and cytotoxic T-lymphocyte-associated proteins. PD-1 is expressed in T regulatory cells (Tregs) which inhibit the proliferation and degranulation of T cells and APC, binding PD-L1 that is also upregulated in TAMs and in other immune cells. The expansion of Tregs is also promoted by immunosuppressive monocytes such as myeloid-derived suppressor cells that exert their effects locally, releasing immunomodulatory cytokines [79,80].

The pathogenic effects of oncometabolites in driving the immunosuppressive state of GBM are also mediated by complex mechanisms that definitely rely on epigenetic deregulation. The depletion of α-KG affects the canonical functioning of the prolyl hydroxylases (PHD) in the hydroxylation of HIF-1α towards its ubiquitination [81]. Consequently, the production of 2-HG indirectly supports the dimerization of HIF-1α and the transcription of hypoxia response-element (HRE). Changes in chromatin structure, especially in histone methylation, acetylation and DNA methylation, are promoted by HRE transcription [82]. The downstream effects of this transcriptional regulation are related to the promotion of several cancer hallmarks including immune escape, such as the production of immunosuppressive cytokines and immunosuppressive myeloid-derived suppressive cells [23,79].

In a recent work by Friedrich and coworkers, it was highlighted that IDH-mut GBM reshapes immune populations, exploiting tryptophan metabolism-mediated immunosuppressive responses via 2-HG [83]. The authors identified subclasses of infiltrating myeloid cells in GBM IDH-mut that longitudinally change during tumor progression, generating an immunosuppressive TME. Surprisingly, infiltrated myeloid cells use 2-HG and tryptophan degradation via the kynurenine pathway and tryptophan 2,3-dioxygenase (TDO) activation leading to a metabolic reprogramming. The main transporters involved in these mechanisms (i.e., LAT1-CD98) mediate tryptophan intake in a dose dependent fashion, leading to IL-10 and TGF-β production [83].

The involvement of tryptophan metabolism in mediating immunosuppression processes was evaluated in another study, which reported tumor-associated macrophage (TAM) recruitment and aryl hydrocarbon receptor (AHR) signaling, but no association with 2-HG production. Indeed, GBM cells have been reported to directly produce l-kynurenine by stimulating AHR-mediated recruitment of TAMs via CCR2 signaling triggered by CCL2 [84]. Furthermore, this study described epigenetic factors with low expression of miR-29b in TAMs able to suppress AHR levels [84]. It has been also shown that high levels of NAD^+^ are generated by the tryptophan and kynurenine metabolisms. Furthermore, a hypomethylation state in GBM has been associated with high NAP^+^ levels, leading to mesenchymal phenotypes and cancer progression [85].

The involvement of epigenetic processes in the immune evasion mechanism of GBM has been identified in another work revealing interesting perspectives linked to a process known as epigenetic immunoediting [86]. First, these findings were not linked to IDH-mut and metabolic alterations’ relationship; engineered GSCs from neural stem cells were used to model the mesenchymal subtype with Nf1/Pten co-deletion and EGFRvIII overexpression. Immune cells have been observed to be the key responsible factor for the remodeling of GBM cell transcriptomics leading to an immunosuppressive TME. Therefore, the mutual connection between tumor and immune cells has been highlighted as, upon attack by the immune system, GBM modifies its transcriptomic profile to enhance an immunosuppressive myeloid-enriched TME [87]. Indeed, the most interesting result was related to the evidence that GBM cells, along with tumor progression, showed the upregulation of genes, such as interferon regulatory factor 8 (IRF-8) and chemokines belonging to the myeloid population, regardless of gene mutations and clonal selections. The maintenance of genomic stability, concomitantly with gene expression changes, has been attributed to epigenetic alterations that have been confirmed with the site-specific DNA methylation of GBM by interferon regulatory factor signaling [86]. Molecular subtyping of patient-derived GSCs revealed decreased levels of DNA methylation associated with a distinct transcriptional profile related to interferon gamma signaling as a dominant feature [86]. Moreover, induction of the mesenchymal phenotype in GBM by macrophages was associated with the production of specific ligands such as the pleiotropic cytokine of the interleukin-6 family Oncostatin M and leukemia inhibitory factor receptors. These are associated with glycoprotein GP130 on GBM cells, in activating STAT3 signaling and a lower expression of colony stimulating factors [88].

It has been reported that glucose transporters were upregulated by hypermethylation and hypomethylation of CpG islands encoding for derlin-3 and caveolin-1, which are a glucose transporter (GLUT) inhibitor and GLUT stimulator, respectively [89]. More broadly, the upregulation of glycolytic enzymes such as pyruvate kinase M1/2 (PKM1/2) and hexokinase 2, mediated by epigenetic mechanisms, leads to an increase in glycolytic metabolism. PKM1/2 was also upregulated by c-myc expression after histone deacetylation, including the action of specific miRNA [90]. PKM2 also binds to histone H3 and phosphorylates histone H3 at T11 upon EGF receptor activation, determining histone H3 at lysine 9 with upregulation of cyclin D1 and c-myc expression [90]. MYC is a key oncogene belonging to transcription factors that coordinate the expression of several genes also involved in PD-L1 regulation [91]. The involvement of c-myc in GBM tumorigenesis, associated with epigenetics and metabolism reprogramming interplay, has been further demonstrated by recent reports [92]. In fact, it has been reported that specific mutations on the telomerase reverse transcriptase (TERT) gene enhance the trimethylation of histone H3 Lys4 (H3K4me3) and the recruitment of the multimeric GA-binding protein A (GABPA) in de novo binding motifs for the E-Twenty-Six transcription factor family members. This process is orchestrated by ERK1/2-dependent phosphorylation of arginino-succinate lyase at Ser417, facilitating the recruitment of GABPA and c-myc to TERT. In this context, H3K4me3 is driven by the inhibition of KDM α-KG-dependent due to fumarate production [92]; in this framework, it is worth noticing that telomere Repeat binding Factors have been implicated in immune escape [93].

GBM glycolytic activity causes low glucose availability in the TME, which determines the exhaustion phenotype in T cells; impaired lymphocyte proliferation, activation and degranulation have been associated with an anti-inflammatory phenotype after lactate exposure [94,95]. The enhancement of glycolytic metabolism is mediated by TGF-β, with TGF-β/Wnt inhibiting the expression of cytochrome C oxidase and resulting in increased glycolysis, which downregulates tumor surface antigens such as HLA-DR, NKG2DL and intercellular adhesion molecule 1, impairing immune cell infiltration and leading to tumor cells escaping from immune surveillance [96]. Acetyl-CoA from pyruvate dehydrogenase provides a precursor for fatty acid and cholesterol synthesis, but it can also modify histone proteins by direct acetylation [29]. In addition, acetyl-CoA is also a substrate with NADPH through the mevalonate pathway to the de novo synthesis of cholesterol. Farnesyl diphosphate synthase belongs to the catalytic reactions to finally generate cholesterol, and it is involved in maintaining the stemness of GBM with prenylation, considered essential for nuclear remodeling [97,98]. Moreover, prenylation has also been associated with promoting immunosuppressive function on T cells and in the regulation of PD-L1 expression [99,100].

The formation, maintenance and recurrence of GBM are mainly governed by GSCs, which can be considered a subset of tumor cells with a significant ability to proliferate and a high self-renewal capacity [101]. GSCs are primarily localized in specific niches, including the so-called immune niche, where they promote signaling pathways that ensure the maintenance of a hypoxic TME, which stimulates the production of cytokines and immunosuppressive factors [102]. Their pre-eminent role in GBM pathogenesis has also been associated with immune suppression and metabolic reprogramming that can be classed under epigenetic regulation [101]. A recent study has shown how maintaining stemness and cell anaplasia in GBM is mediated by interconnected epigenetic and metabolic processes. In this study, Kosty and coworkers investigated the role of Serpine1 mRNA-binding protein 1 (SERBP1), an oncogene RNA-binding protein, that has been found overexpressed in several tumors, including high-grade glioma [103]. Their study demonstrated that the expression of this protein is essential for maintaining the undifferentiated state of GBM cells by contributing to cell renewal and stemness. In particular, it was observed that SERBP1 regulates the metabolism of folate substrates by the interconnected serine biosynthesis and one-carbon (1C) cycle metabolic pathway, leading to additional metabolites such as cysteine and methionine. The first is involved in GSH production, whereas the second is associated with epigenetic processes via H3K27me3 levels, upregulating genes for neurogenesis and neuronal differentiation [104]. Although the authors analyzed effects related to the maintenance of tumor progression, they did not evaluate the correlation to the immune evasion processes. However, the gene modulation-controlling cancer epigenome through methylation was also associated with PI3K/AKT signaling, which has been largely recognized as a key player in immunosuppression. Indeed, it has been reported that T cell apoptosis was induced under PI3K control by the co-stimulatory molecule B7-homologue 1 that is overexpressed in PTEN loss tumor subtypes [105].

In the epigenetic landscape, miRNAs certainly play a role of primary significance in GBM progression, involving several hallmarks of cancer [106]. However, there are few references in the literature connecting them to GBM immunometabolism. Rather, the role of miRNAs is often singly analyzed in metabolic reprogramming and immunosuppression processes, in which major effort is needed to understand the touchpoints. Certainly, the direct gene expression modulation of miRNA-mediated metabolic enzymes has downstream consequences with immunosuppressive effects, as in the case of miR-326 targeting PKM2 [107]. In addition to the hypomethylation processes of genes encoding glycolytic enzymes, miRNAs have been described to act on c-MYC and on the PI3K/Akt pathway, leading to glucose transporters’ accumulation, and glutamine levels as well, supporting the Warburg effect [108,109,110]. However, the expression of glycolytic transporters and enzymes in GBM were also indirectly regulated by miRNA, targeting receptors’ tyrosine kinases [111,112]. As for immunosuppression, several key miRNAs may be regulating multiple mechanisms of both innate and adaptive immune responses [113].

A long list of miRNA expression profiles can be made in relation to the immunosuppression process they are controlling. However, this goes beyond the scope of this review which, among the epigenetic changes in relation to metabolism, wants to better highlight those related to chromatin remodeling rather than to non-coding sequences.

### 3.1. ROS Contribution to Immunosuppressive State

Mitochondrial dysfunction and oxidative stress are concomitant events in cancers, mainly attributed to the strong dependence on less energy-deriving pathways. Metabolic alterations in cancer contribute to the loss of redox balance between reactive oxygen species (ROS) elimination and production, creating a TME that supports tumor progression. Impaired mitochondrial metabolic capacity in GBM is strongly correlated with ROS production and antioxidant defense dysfunction [4]. During mitochondrial oxidative phosphorylation and electron transfer chain, the alteration of the flow rate of the respiratory chain increases the formation of superoxide radical and the consequent formation of the hydroxyl radical [114,115]. In this regard, cells may act with detoxification systems such as superoxide dismutase or glutathione peroxidase, converting GSH into its oxidated form to produce oxygen and water from hydrogen peroxide. In GBM IDH-mut, the dysregulation of TCA, towards the production of 2-HG, not only drives cells toward aerobic glycolysis but also interferes with the electron transport chain, altering mitochondrial physiology [116]. In addition to these effects, the NADPH consumption for IDH-mut activity affects GSH restoring and suppresses the chain of detoxification systems interfering with antigen presentation and T cell proliferations [117,118,119]. Therefore, the source of 2-HG increases oxidative stress and ROS in cancer cells, which encourages tumor cell growth [120]. These characteristics induce, on the one hand, the accumulation of ROS that might further stimulate tumor development due to the increase in genetic instability; on the other hand, tumor cells’ vulnerability to oxidative damage arises with decreasing GSH levels [121]. Increased oxidative stress in IDH-mut forms could explain the weakness of low-grade gliomas, although additional mechanisms related to IDH may be implicated in tumor viability. Indeed 2-HG has also been shown to function as an ATP synthase inhibitor and interfere with mTOR signaling, leading to a decrease in tumor growth and cell viability, whereas IDH-wt forms correlate to older patients with shorter than median survival, due to additional mutations such as higher EGFR amplification [122,123].

GBM response to mitochondria stress can also be examined in relation to the mitohormesis mechanism. More precisely, the term mitohormesis indicates a biological response to mitochondrial stress which can also lead to an increment in health and viability [124]. Indeed, mitohormesis enhances the effectiveness of adaptive metabolic strategies that produce acquired resilience leading to an enlarged healthspan, particularly improving metabolism and the immune system [125]. The positive benefits exerted by mitohormesis require a coordinated communication with the nuclear processes based on the redox-activated transcription factor [126]. Moreover, upon oxidative stress and hypoxia, which are common traits of GBM, the transcription factor nuclear factor erythroid-derived 2-like 2 (NRF2) is upregulated, linking mitochondrial dysregulation and epigenetic changes [127,128,129]. Indeed, oxidative stress-induced NRF2 nucleus translocation to bind antioxidant response elements (ARE) encodes proteins involved in response to stress, such as injury, inflammation and free radicals’ production, including GSH synthesis [130,131]. Enzymes participating in the PPP are also expressed due to the epigenetic regulation of NRF2 [132]. The NRF2 involvement in epigenetic changes may be found in its cooperation with cobalamin metabolism and the D_4_ receptor on the regulation of the epigenetic state in both GBM and myeloid cells [133]. Furthermore, the lack of ubiquitination of NRF2 triggers the transcriptional cascade of the vitagene network, including redox sensitive genes, such as heme oxygenase-1 (HO-1), heat shock proteins (Hsps), thioredoxin (Trx) and SIRT1 [134,135]. In recent years, the vitagene system has emerged as a potential target, as it has been shown to have a high neuroprotective power. Therefore, the discovery of molecules capable of activating this system may represent a new therapeutic strategy to limit the consequences induced by oxidative stress, such as tumor progression [136]. HO-1 has been reported to maintain an immunosuppressive TME, this being expressed as monocyte and TAMs that suppress the antitumor CD8^+^ T cell effector [137,138,139]. Interestingly, HO-1 expression was demonstrated to educate myeloid transcriptional and epigenetic programs leading to the control of PD-L1, PD-L2 and MERTK expression [140]. Furthermore, the immunosuppressive activity of bone marrow-derived macrophages in the glioma TME was abolished after HO-1 inhibition, restoring T cell proliferation. These results were both also associated with tryptophan metabolism, since myeloid HO-1 activity significantly increases IDO1 and to PD-L1 expression through the phosphorylation of STAT3 [141]. Hsps were involved in several mechanisms of proteostasis, including the host immune system evasion; indeed, T cell activation through T cell CD3-zeta downregulation is inhibited by HSP10 [142]. There are no direct correlations between SIRT1 and the epigenetic modifications that regulate immune escape in tumors. However, SIRT1 is involved in a multitude of effects related to histone and non-histone deacetylation that may include signal transduction and gene transcription suppressing the TME. It has been reported that in B cells, the activation-induced cytidine deaminase suppresses SIRT1 expression [143]. Moreover, IL-2 production was induced by c-Jun transcription factor inactivation mediated by SIRT1 [144]. Vice versa, it has also been reported that SIRT1 activates the adaptive response and differentiation of Th2 cells; there is also evidence indicating the enhancement of Treg acting as immune suppressive and inducing allograft tolerance [145].

In addition to ROS, reactive nitrogen species (RNS) also play a significant role in redox homeostasis alteration, contributing to further pathophysiological effects for TME supporting. RNS derive from nitric oxide (NO), a highly reactive molecule synthetized by NO synthase (NOS). Under physiological conditions, NO acts as a second messenger with the activation of cyclic guanosine monophosphate, generating multiple signaling pathways and downstream effects for neurotransmission [146]. NO plays an important role in the regulation of oxidative stress by providing neuroprotection with the production of antioxidants. However, when the homeostatic equilibrium is lacking, the effect of NO becomes cytotoxic and, in the presence of free radicals and inflammatory conditions, generates RNS leading to protein nitration and cell damage [147]. Most studies are limited to analyzing the pro- or antitumor effects of NO in GBM rather than its role in relation to immunometabolism under epigenetic control [148]. The catalytic activity of JmjC-domain, containing histone demethylases, has been found to be inhibited by NO exposure in cancer cells, fostering an oncogenic phenotype [149]. Furthermore, the emerging correlations with epigenetic alterations have led to the definition of epigenetics as the third pillar of NO signaling, in addition to its role in soluble guanylate cyclase production and protein nitration [150,151]. The gap in mechanistic interpretation of transcriptional programs in response to oxidative stress, including ROS and RNS exposure, may be filled by the knowledge of epigenetic changes. Understanding these processes could provide further insights into GBM immunometabolism.

**Figure 2:**
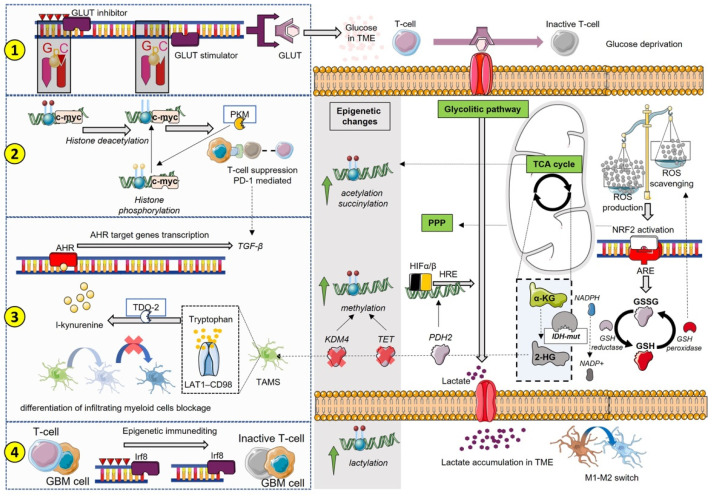
Several paths are involved in metabolism and epigenetics’ interplay for immunosuppression. We summarized four different ways that should be recognized as interconnected rather than separate from each other. Starting from (**1**), the CpG islands control the expression of enzymes and factors involved in several metabolic pathways; therefore, the state of hypomethylation and hypermethylation can enhance or reduce specific metabolic pathways. For instance, methylation of GLUT inhibitors determines an increase in glucose intake, depleting the TME, where myeloid cells will not be able to perform their functions. TCA break determines an increase in lactate and a TME acidification that, besides increasing the processes of lactylation, determines mechanisms of immunosuppression including reshaping macrophages’ phenotype. (**2**) shows the direct involvement of epigenetic-mediated c-myc gene expression in association with PKM production and PD-1/PD-L1 immunosuppression mechanisms. (**3**) describes the contribution of tryptophan metabolism in 2-HG-induced TAMs, which results in the activation of AHR signaling with the production of interleukins and immunosuppressive cytokines, such as TGF-β. The production of 2-HG determines methylation changes linked to the blockade of TET and KDM4, as well as to the modulation of HIF mediated by the lack of PHD activity. (**4**) illustrates the effects of immune and tumor cells’ interplay inducing the so-called epigenetic immunoediting that re-educates and hijacks the transcriptional programs of the tumor towards an immunosuppressive phenotype. In this broad scenario, the alterations of the mitochondrial respiration chain must be taken into account. ROS production activates the response of genes to oxidative stress, which includes NRF2 signaling with the expression of genes and factors involved in metabolic reprogramming and immunosuppression. Among these, the production of GSH certainly plays an important role in detoxification processes that may be hindered by NADPH consumption for IDH-mut activity. Functional depletion of GSH has also an impact on antigen presentation and T cell proliferation.

Taken together, we reported an integrated vision of immunometabolism that shows how immune mechanisms’ and metabolic pathways’ interplay can be the cause or the effect for epigenetic changes in a complex system where metabolites can be either the products or the source of post-translational modifications driving the immunosuppressive state for GBM progression (Figure 2).

### 3.2. Limitations and Challenges of Immunotherapy

Phase II and III clinical trials have been performed for immune checkpoint inhibitors in both newly diagnosed and recurrent GBM. Nivolumab is one of the most common FDA-approved monoclonal IgG4 antibodies targeting the PD-1 receptor, which has been tested in combination with bevacizumab and with temozolomide. It was also tested comparing gene promoter profile methylation of O(6)-methylguanine DNA methyltransferase (MGMT) [152,153]. Pembrolizumab is also a PD1 receptor inhibitor, that was tested in phase I/II for recurrent GBM and phase III for neoadjuvant GBM, reporting both local and systemic antitumor immune response enhancement. Despite the local anti-tumor immune response increase observed in GBM patients treated with this anti-PD1 receptor, overall survival improvements have not been reported to declare the success of this drug [154,155,156]. Other FDA-approved immune checkpoint inhibitors, such as atezolizumab, were directed to target PD-L1. Atezolizumab efficacy for GBM is still under investigation, although promising results have been observed for peripheral CD4^+^ T cells and in evaluating the correlation of hypermutation phenotypes with tumor mutation analysis [157].

Immune checkpoint inhibitor limitations may lay in several potential issues that make challenging the current approaches for GBM. First, the genomic heterogeneity of GBM is crucial in determining the efficacy of immunotherapies, since specific mutations such as PTEN, MAPK pathway alterations, germline DNA polymerase epsilon and broad mutation, such as somatic mutations, microsatellite instability and tumor mutational burden, seem to acquire prognostic and predictive values [158]. If on the one hand, the stratification of GBM types can be beneficial in guiding the treatment personalization, on the other hand, the high tumor heterogeneity hinders the immunotherapy approaches. Some limitations, though, which can be broadly referred to as several pharmacological treatments, should be also mentioned in the case of immunotherapy. These are related to the pharmacological issues for achieving the adequate doses based on the CNS toxicity that may also be associated with immune response or a misdirected immune-mediated injury increasing intracranial pressure [159]. In conclusion, the Immunotherapy Response Assessment in Neuro-Oncology criteria have a key role in interpreting radiographic endpoints that, after immunotherapy, may be critical to confirm the effectiveness of the treatment, especially to discriminate the radiologic features of tumor progression from the inflammatory response induced by an effective anti-tumor immune response [160].

The transitions from preclinical evaluation to the clinical development of potential immunotherapy require appropriate preclinical models. The most commonly used over the years in the immunotherapy setting are represented by syngeneic mice, which show several advantages, including their logistically easy application, but also show limitations in matching directly with human clinical disease and outcomes [161]. This limitation is overcome by the use of transgenic mouse models or genetically engineered mouse models which reproduce a faithful stromal biology, although the few neo-antigen formations might hinder the immune oncology evaluation [162]. Cell line- and patient-derived xenografts have been currently applied for immune oncology studies, overcoming major limitations of syngeneic and genetically engineered mouse models; however, the poor predictive values, the heterogeneity loss and the difficulty in evaluating immune-mediated responses are critical points [163].

The issues listed in the field of immunotherapy, if retained within the epigenetics and metabolic reprogramming interplay, acquire further complexity. In this regard, preclinical studies should be addressed to the pathophysiological mechanisms’ evaluation in response to the novel and currently targeted therapies for GBM, including antiangiogenic agents and kinase inhibitors combined with immune checkpoint therapy, considering the tumor subtype and biological profile.

## 4. Conclusions

Immunotherapy represents a promising strategy for the treatment of GBM. However, the different clinical approaches and attempts encounter significant obstacles represented by the processes of immunosuppression, which therefore need to be further investigated.

In the intricate TME of GBM, metabolic reprogramming, oncometabolite production and oxidative stress support immunosuppression. Epigenetic changes establish a complex interaction with metabolic rewrite actors orchestrating a condition promoting immunosuppression, including the production of specific metabolites that ultimately affect chromatin states. The correlation between epigenetics and metabolism in ensuring an immunosuppressive phenotype is strongly mediated by IDH activity with downstream and upstream processes and effects. However, many other immunosuppressive mechanisms are activated, including the prominent role of the stress response pathways, thus suggesting the need to explore additional factors underlying immunosuppression in the complex TME of GBM in an effort to highlight new drug targets. Metabolism, immunosuppression, and epigenetic regulation are undoubtedly macroscopic and complex areas. Each field contains a multiplicity of cellular and molecular mechanisms whose relational analysis is challenging. Many processes are still unclear, but remarkable research perspectives have been progressively emerging. Among them, the role of GSCs in the interplay between immunosuppression and metabolic reprogramming may reveal interesting scenarios since they play a prominent role in the pathogenic processes of GBM. Further efforts will be needed to expand this research field which could provide better therapeutic strategies for GBM treatment.

## Data Availability

Data is contained within the article.

## Abbreviations

α-KG	α-ketoglutarate
AHR	Aryl hydrocarbon receptor
APCs	Antigen presenting cells
ARE	Antioxidant response elements
DNMT	DNA methyltransferase
EGFR	Epidermal growth factor receptor
GABPA	GA-binding protein A
GLUT	Glucose transporter
GBM	Glioblastoma
GSCs	Glioblastoma stem cells
GSH	Glutathione
H3K4me3	Trimethylation of histone H3 Lys4
HIFs	Hypoxia inducible factors
HO-1	Heme oxygenase-1
HRE	Hypoxia response-element
Hsps	Heat shock proteins
IDH	Isocitrate dehydrogenase
IDH-mut	IDH-mutated
IDH-wt	IDH-wild type
IL-10	Interleukin-10
IL-23	Interleukin-23
IRF-8	Interferon regulatory factor 8
JHDM	Jumonji-domain histone demethylase
KDM	Lysine-specific demethylase
LSD1	Demethylation functions of histone demethylase 1
miRNA	MicroRNA
NO	Nitric oxide
NOS	NO synthase
NRF2	Nuclear factor erythroid-derived 2-like 2
PHD	Prolyl hydroxylases
PKM	Pyruvate kinase M1/2
PPP	Pentose phosphate pathway
RNS	Reactive nitrogen species
ROS	Reactive oxygen species
SAM	S-adenosyl methionine
SIRT1	Sirtuin
TAMs	Tumor-associated macrophages
TCA	Tricarboxylic acid
TDO	Tryptophan 2,3-dioxygenase activation
TERT	Telomerase reverse transcriptase
TET	Ten-eleven translocation enzymes
TGF-β	Transforming growth factor-beta
TME	Tumor microenvironment
Tregs	Regulatory T cells
Trx	Thioredoxin
2-HG	2-hydroxyglutarate
